# Preoperative low scores of Life Satisfaction Rating predicts poor outcomes after total knee arthroplasty: a prospective observational study

**DOI:** 10.1186/s13018-020-01668-9

**Published:** 2020-04-15

**Authors:** Kaiyuan Liu, Dong Yang, Pengfei Zan, Aoyuan Fan, Zhi Zheng, Wenwei Jiang, Guodong Li

**Affiliations:** 1 Department of Orthopedic Surgery, Shanghai Tenth People’s Hospital affiliated to Tongji University, 301 Yanchang Rd, Jingan District, Shanghai, 200072 People’s Republic of China; 2 Department of Orthopedic Surgery, Shanghai General Hospital affiliated to Jiatong University, No.100 Haining Road, Hongkou District, Shanghai, 200080 People’s Republic of China; 3 Department of orthopedic surgery, Jinshan Hospital affiliated to Fudan University, Shanghai, People’s Republic of China

**Keywords:** Total knee arthroplasty, Satisfaction, Psychological, Life Satisfaction Rating

## Abstract

**Background:**

Despite the continued improvement in the surgical techniques during primary total knee arthroplasty (TKA), literatures indicate that up to 10 to 20% patients are not satisfied with their outcomes. Psychological factors in this dissatisfaction are yet to be clearly identified. The aim of this study is to develop a method to assess whether the patient’s current mental state is suitable enough to accept a TKA surgery.

**Methods:**

Preoperative demographic and clinical data of 532 patients who underwent TKA were prospectively obtained from January 2012 until December 2016. We recorded the scores evaluated by SF-36 questionnaire and Western Ontario and McMaster Universities Osteoarthritis Index (WOMAC) preoperatively and 1 year postoperatively. Preoperative Life Satisfaction Rating (LSR) is emphatically evaluated.

**Results:**

Poor preoperative score of LSR was a significant predictor of dissatisfaction after TKA. Patients with low LSR reported significant pain and stiffness, although there was no remarkable effect on functionality of the replaced joint. The results also showed that age and BMI were not strong predictors of satisfaction in TKA.

**Conclusion:**

Our outcomes can help clinicians evaluate whether a patient’s current mental status is favorable for TKA. If patients have extreme low scores of LSR (less than 10), a psychological intervention should be recommended for better satisfaction following a TKA surgery. This would also allow surgeons to individually assess the risks and benefits of surgery.

## Introduction

Total knee arthroplasty (TKA) is the most effective surgical treatment for improving physical function and alleviating pain in end-stage osteoarthritis [1–4]. Patient’s satisfaction is now being considered as an important aspect in the evaluation of a TKA outcome [5]. Despite the rapid advancement in the surgical technology, facilities, and prosthesis, studies indicate that patient’s satisfaction with TKA is only 68 to 93% [3, 6–8]. Edwards et al. reported that at least one fourth of the patients experienced substantial pain and functional limitation 1 year after TKA, whereas without any clear physical or functional reasons [9]. Patient-perceived outcomes are presently recommended as a core component in the evaluation of TKA surgery [10]. Recently, psychological determinants, such as anxiety, depression, and expectancy, have been increasingly identified as risk factors for poor patient-perceived outcomes [11–14]. Gong et al. [15] retrospectively evaluated the relationship between patients’ diverse personalities and clinical outcomes after TKA and emphasized the importance of preoperative attention to patient personality. Likewise, Giurea et al. [16] found a significant association between personality traits and TKA outcomes by using the Freiburg Personality Inventory (FPI-R). Putting together, baseline mental health may affect patients’ satisfaction levels, their long-term perception of pain, as well as their motivation to return to the desired level of functionality.

Currently, the most related psychological state and appropriate methods to assess a preoperative patient’s status are yet to be determined [17]. It is still unclear under what conditions psychological intervention should be performed for achieving better satisfaction after TKA. As we know, the Life Satisfaction Rating (LSR) scale proposed by Neugarten and Havighurst in 1961 [18], a multidimensional measure representing the complexity of psychological wellbeing, has been widely used since its publication [19, 20]. The hypothesis of this study is that the outcome of LSR is a significant predictor of TKA. Thus, we conducted this prospective observational study to identify a comprehensive evaluation tool to determine whether the patient’s current mental status is suitable to accept TKA and whether preoperative psychological intervention would be necessary. We hypothesized that the LSR scoring system is a useful tool to evaluate a preoperative patient’s mental status, and low preoperative LSR would predict poor outcomes following a TKA procedure.

## Material and methods

### Study protocol

This was a prospective observational study conducted by the department of orthopedic surgery at the institution of Shanghai Tenth People’s Hospital. Patients scheduled for TKA were required to make an appointment 3 to 7 days before the operation. Our first questionnaire was delivered to the patients at the time of making the appointment; the items of the questionnaire included age, sex, body mass index (BMI), comorbidities (based on American Society of Anesthesiologists [ASA] grade), Western Ontario and McMaster Universities Osteoarthritis Index (WOMAC), and Short-Form Health Survey of 36 questions (SF-36). In addition, during the appointment, patients had counseling by a team of professional psychologists in the department of psychiatry to administer the LSR. Routine outpatient follow-up was conducted at 1 month, 3 months, 6 months, and 1 year postoperatively. ROM has recovered to a better condition and thus was recorded at postoperative 6 months, and satisfactory evaluations were conducted at postoperative 1 year, which included WOMAC and SF-36 as well as satisfaction degree.

The study protocol was approved by the Ethics Committee of our institution, and all the patients were aware of the purpose and procedure of our study and signed consents to participate.

### Patients

The age of the 532 participants in our study ranged from 42 to 83 years. All patients had undergone primary TKA between January 1, 2012, and December 1, 2016. The decision for primary TKA was taken completely based on the clinical indications and contraindications. The inclusion criterion for the study was unilateral, primary TKA recommended only for osteoarthritis. Exclusion criteria were rheumatoid arthritis, infection after TKA, improper alignment caused by extra articular deformity, prosthesis-related complications, psychopathology history, or mental disorder. Of the 549 patients who met the inclusion criteria of the study, we excluded 7 patients who were diagnosed with cancer within 1 year of TKA and 10 more patients with existent psychopathology or mental disorder(s). The baseline data of the 532 patients are summarized in Table 1. Postoperative alignments were regarded as acceptable if they were within 0° ± 3 of the mechanical axis.

**Table 1:** Basic data of satisfied and dissatisfied patients [mean ± standard deviation (range)]

Factors	Satisfied	Dissatisfied	*p* value
Number	446(83.8%)	86(16.2%)	–
Age (years)	65.3 ± 6.3(52–83)	66.8 ± 6.9(54–83)	0.055
Sex (M/F)	117/329	23/63	0.922
BMI (kg/m^2^)	26.4 ± 4.7(19–45)	27.3 ± 5.1(20–44)	0.093
Comorbidity (ASA ≥ 3)	29.4%	40.1%	0.043*

*BMI* body mass index, *ASA* American Society of Anesthesiologists

*Statistically significant difference between groups

### Surgical procedure

**Fig. 1:**
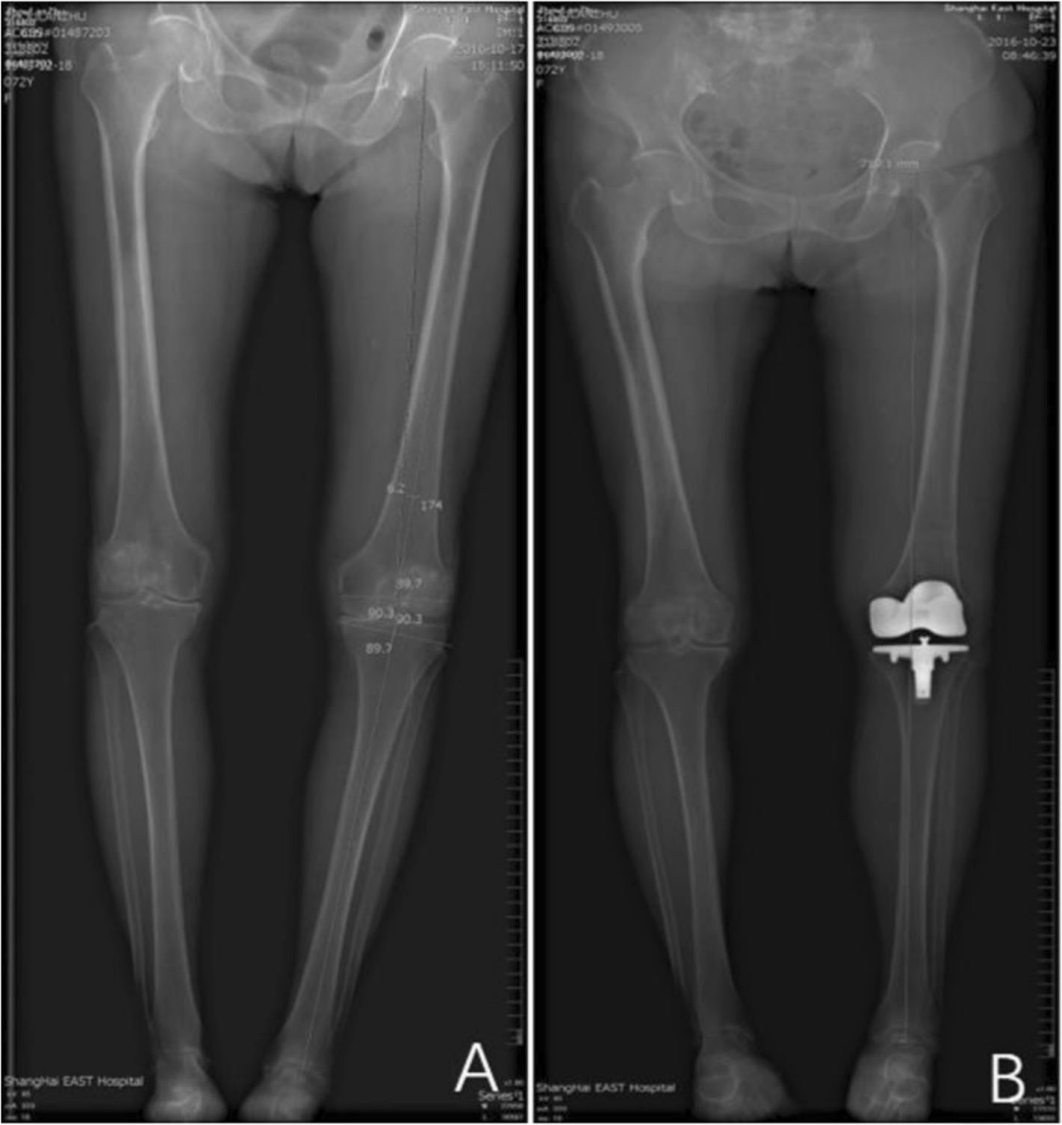
Pre- and post-operative long leg standing anteroposterior X-ray images. **a** Pre-operative X-ray shows the distal lateral femur angle, the proximal medial tibial angle, and the mechanical axis. **b** Postoperative X-ray shows a neutral mechanical axis

Before the surgery, a long-leg standing radiograph was acquired in the anteroposterior view. The same orthopedic surgeon analyzed the distal lateral femur angle, the proximal medial tibial angle, and the mechanical axis, which guided the intraoperative alignment (Fig. 1a). We routinely administered preoperative antibiotics (second-generation cephalosporin). All surgeries were performed by a senior surgeon (Dr. Guodong Li, who is the corresponding author of the study). A standard TKA procedure was performed under general anesthesia using a posterior cruciate-substituting cemented prosthesis (GenesisII Oxinium; Smith & Nephew, Memphis, Tennessee) with a longitudinal midline incision and a medial parapatellar approach. Before wound closure, a diluted 60 ml cocktail containing a single dose of morphine, bupivacaine, and betamethasone was administrated for pain control. Patients received postoperative oral non-steroidal anti-inflammatory medication with celecoxib capsules (Pfizer) if necessary. Drainage tube was removed 24 h after surgery. Three days after the surgery, another long-leg standing radiograph in the anteroposterior view was obtained to confirm proper joint alignment (0° ± 3 of the mechanical axis) (Fig. 1b). All patients were mobilized using a routine physiotherapy protocol on the first postoperative day after removal of the drainage tube.

### LSR

LSR was used to evaluate the patient’s current mental status in our research. In this index, five components of life satisfaction were identified: (a) zest versus apathy—the degree of involvement in activities, with other persons, or with ideas; (b) resolution and fortitude—the extent that persons take responsibility for their own lives; (c) congruence—the extent to which life goals were achieved; (d) self-concept—the person’s concept of self, physically, psychologically, and socially; and (e) mood tone—whether the person holds optimistic attitudes and happy feelings. Each component contains five subscales (A to E), and each subscale is graded from 1 to 5 points. For example, subscale A has five options to describe how enthusiastic the assesse is with his/her present life. Five points implies passion in life, while 1 point indicates indifference towards the surrounding occurrences [18].

### SF-36 Health Survey Questionnaire

Since its introduction in 1980s, SF-36 has been the most widely used clinical instrument for the evaluation of health-related quality of life. It consists of two main components: physical component summary (PCS) and mental component summary (MCS). Higher scores predict better health status [21].

### WOMAC Index

This is the most common self-administered tool used to assess the health status of osteoarthritis patients. It consists of 33 items that evaluate pain (5 questions), stiffness (2 questions), and degree of disability in daily life activities (17 questions). Each question has five subscales graded from 0 point (best score; never or none) to 4 points (worst; extreme or always) [22].

### Satisfaction

The domain related to satisfaction contained two questions: (1) “Overall, are you satisfied with the results of your primary TKA? Yes or No.” (2) “Describe how satisfied you are with scores from 0 to 100, the higher scores you fill in, the more satisfied you are.” [23].

### Statistical analysis

Continuous variables are presented as mean ± SD, and categorical variables are presented as percentages. The differences between the SF-36 and WOMAC subscale scores in the satisfied and dissatisfied groups were determined using the *t* test. The satisfaction rate and comorbidity were compared using the chi-square test. A one-way analysis of variance was performed to compare the postoperative subscale scores of LSR, SF-36, and WOMAC in the four groups. A *p* value of < 0.05 was considered a statistical difference. All statistical analyses were performed using SPSS, version 17.

## Results

Of the 532 TKAs, 392 were performed on female patients and 140 on male patients. The satisfied group comprised 446 (83.8%) patients and the dissatisfied group was constituted of 86 (16.3%) patients. The average age of the patients was 65.3 years, the average BMI was 26.4 kg/m^2^, and incidence of comorbidity was 29.4%; these corresponding values were 66.8 years, 27.3, and 40.1% in the dissatisfied group, with no significant differences found between groups. Postoperative radiographic evaluation showed that the main objective outcome of mechanical axis (Fig. 1b) did achieve desired target between the satisfied and the dissatisfied groups, and therefore, the differences between the two groups precluded the effect of any operational factors.

Preoperative data in the satisfied and dissatisfied group were summarized in Table 2. Preoperative LSR and SF-36 PCS scores were significantly better in the satisfied group (*p* < 0.05), and the WOMAC pain and functional scores were also significantly less in the satisfied group (*p* < 0.05), whereas no significant differences were detected regarding of SF-36 MCS and WOMAC stiffness scores.

**Table 2:** Preoperative data of satisfied and dissatisfied patients (mean ± standard deviation)

Factors	Satisfied	Dissatisfied	*p* value
Number	446	86	–
LSR	18.5 ± 3.8	15.4 ± 4.1	0.001*
SF-36 PCS	32.3 ± 9.6	30.0 ± 10.1	0.044*
SF-36 MCS	51.6 ± 12.2	52.9 ± 12.2	0.393
WOMAC pain	10.2 ± 4.0	11.2 ± 3.8	0.038*
WOMAC stiffness	4.7 ± 0.7	4.6 ± 0.8	0.134
WOMAC function	30.8 ± 9.2	33.3 ± 9.2	0.022*

*SF-36* short-form health survey of 36 questions, *PCS* physical component summary, *MCS* mental component summary, *WOMAC* Western Ontario and McMaster Universities Osteoarthritis Index

*Statistically significant difference between groups

**Fig. 2:**
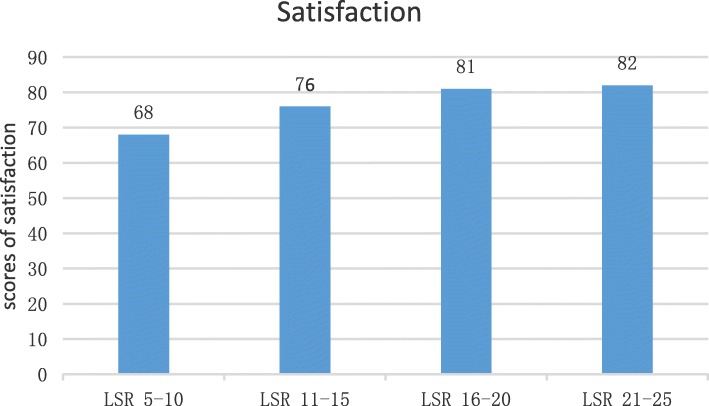
Scores of satisfactions among the four subgroups

As shown in Table 3, among the four groups classified by preoperative LSR (group 1, LSR 5–10; group 2, LSR 11–15; group 3: LSR 16–20; and group 4: 21–25), the group with the lowest LSR score (group 1) had significantly poorer outcomes than the other three groups in terms of SF-36 MCS score, WOMAC pain, and WOMAC stiffness. Further subgroups analysis showed that there was no significant difference between group 3 and group 4; group 1 and group 2 had average postoperative satisfaction scores of only 68 and 76 respectively, which are much lower than those evaluated in group 3 and group 4 (Fig. 2).

**Table 3:** Preoperative and postoperative outcomes among four subgroups classified by LSR scores (mean ± standard deviation)

LSR	5–10	11–15	16–20	21–25	*p* value
Number	12	108	314	98	–
Satisfaction (%)	50.0	82.4	84.7	84.7	0.016*
**Pre-op WOMAC**					
WOMAC pain	11.1 ± 1.5	9.5 ± 2.0	8.5 ± 1.8	8.3 ± 1.9	0.001*
WOMAC stiffness	4.8 ± 0.6	4.7 ± 0.7	4.6 ± 0.7	4.6 ± 0.8	0.386
WOMAC function	32.2 ± 3.9	32.3 ± 4.7	31.5 ± 6.0	31.8 ± 5.2	0.625
**Pre-op SF-36**					
SF-36 PCS	27.3 ± 8.7	31.4 ± 11.2	31.9 ± 9.5	31.9 ± 9.9	0.450
SF-36 MCS	51.8 ± 6.9	52.2 ± 8.2	52.4 ± 8.5	52.3 ± 9.1	0.993
**Post-op WOMAC**					
WOMAC pain	6.8 ± 1.6	5.3 ± 2.0	4.2 ± 1.8	3.9 ± 1.8	0.001*
WOMAC stiffness	1.8 ± 0.4	1.4 ± 0.6	1.2 ± 0.5	1.1 ± 0.7	0.001*
WOMAC function	19.1 ± 3.9	18.9 ± 4.9	17.6 ± 6.0	18.1 ± 5.1	0.213
**Post-op SF-36**					
SF-36 PCS	41.3 ± 9.5	46.5 ± 11.3	46.7 ± 9.5	47.1 ± 9.8	0.288
SF-36 MCS	49.4 ± 7.4	55.9 ± 8.3	54.4 ± 8.5	53.5 ± 9.5	0.046*
Post-op ROM	115.9 ± 18.4	121.3 ± 13.4	119.3 ± 13.8	118.1 ± 12.8	0.287
**Change in WOMAC**					
WOMAC pain	4.3 ± 0.9	4.2 ± 0.8	4.3 ± 0.9	4.4 ± 0.9	0.553
WOMAC stiffness	3.0 ± 0.7	3.2 ± 0.9	3.4 ± 0.9	3.5 ± 1.0	0.097
WOMAC function	13.1 ± 1.4	13.4 ± 2.7	13.9 ± 3.2	13.6 ± 4.0	0.546
**Change in SF-36**					
SF-36 PCS	14.0 ± 2.4	15.1 ± 2.0	14.8 ± 2.9	15.2 ± 3.0	0.340
SF-36 MCS	2.4 ± 0.7	3.6 ± 0.7	2.0 ± 0.9	1.2 ± 0.9	0.001*
Satisfaction scores	68 ± 6.4	76 ± 8.9	81 ± 8.6	82 ± 6.1	0.001*

*LSR* Life Satisfaction Rating, *SF-36* short-form health survey of 36 questions, *PCS* physical component summary, *MCS* mental component summary, *WOMAC* Western Ontario and McMaster Universities Osteoarthritis Index

*Statistically significant difference between groups

## Discussion

The recent interest in patient-centered care has prompted several investigations on the patient perspectives regarding TKA outcome [24, 25]. Several studies have indicated that not all but only 68~93% of the patients undergoing TKA are satisfied. In this study, we obtained a patient satisfaction rate of 83.8% at postoperative 1 year after TKA, which is comparable to the rates reported previously [3, 6–8].

Both internal and external factors have been recognized as determinants of patient’s satisfaction [12, 26]. In case of TKA, the external factors associated with patient’s satisfaction include anesthesia, postoperative pain management, surgical technique, implant type, and postoperative rehabilitation [27]. Considering that the surgical procedure, operating surgeon, and the rehabilitation guidance were the same for all patients, we assumed that it was the multiple internal factors that affect the patients’ satisfaction instead of external factors in the cohort. The most important finding of the present study was that the outcomes of LSR was a significant predictor of satisfaction with TKA; we found that patients with low scores of LSR had more pain and stiffness during joint movement, but no significant difference in terms of function (WOMAC function). That means there is a group of people remaining dissatisfied after successful TKA. Our results were consistent with those of other qualitative studies [12, 26] showing that mental health is associated with patient’s satisfaction and clinical outcome after TKA.

Previous studies [12, 26] focused mainly on the associations between psychological factors and satisfaction after TKA, while our study intended to work on identifying the conditions under which a TKA should be put off and a psychological intervention may be helpful. Some investigators have recommended that psychological intervention be administered according to patient’s personality [15, 16]. However, the practical application of this recommendation has certain limitations. On one hand, the outcomes of personality determination do not reflect the patients’ recent psychological states; on the other hand, personality develops over a long period, and psychological intervention has little effect on an individual’s personality. As a result, surgeons cannot evaluate whether the intervention is useful. Moreover, estimating a patient’s mental status according to individual mental factors or incorporating these psychological variables into the prediction of the TKA outcomes is not practical. It would not be reasonable to evaluate to the patient with respect to various mental characteristics to rule out patients who are not mentally prepared to undergo primary TKA.

Our results showed that the scores of LSR are not sensitive, but highly specific in the assessment of patient’s satisfaction with TKA. According to the levels of LSR, we found a significant difference in SF-36 MCS, WOMAC pain, and WOMAC stiffness in postoperative 1 year (Table 3). It was obvious that the group with the lowest LSR score had much lower SF-36 MCS and much higher WOMAC pain and WOMAC stiffness than the other three groups, whereas levels of SF-36 PCS and WOMAC function were similar. In particular, the improvement for the WOMAC function and SF-36 PCS has no significant difference across the 4 groups, then the lower satisfaction rates in the low LSR group can be attributed mainly to a systemic difference between the groups where despite similar clinical outcomes, the low LSR patients continue to rate their satisfaction lowly. This informs that patients with extremely low scores of LSR have more strong subjective feelings such as pain or stiffness despite having similar clinical status as others.

Nevertheless, we do not intend to suggest that low scores of LSR are a contraindication to perform TKAs since patients stand to benefit significantly from surgery in terms of their improvement of function. However, we do recommend that patients with low LSR scores should undergo a psychological intervention to inform them regarding the outcome to be expected. Our findings were in accordance to those published literatures [13, 28, 29]. A systematic review and meta-analysis [28] found that preoperative pain catastrophizing, mental distress, symptoms of anxiety or depression, and somatoform disorders appear to adversely affect pain and function; thus, they recommended preoperative psychological support may be necessary in some individuals. Yasser et al. [29] conducted a cross-sectional study to examine psychological traits in patients waiting for TKA surgery; they suggested that psychological health should be better assessed and treated before surgery to help reduce preoperative dysfunction and improve postoperative outcomes following TKAs.

In general, LSR encompasses several domains that affect the mental status. A previous study found that some independent factors, such as work status, living alone, social support, and comorbidities, have considerable impact on TKA outcomes [30]. In fact, apart from the degree of disability, retirement status and socioeconomic status were found to influence the scores of LSR [27, 31]. A recent study showed that waiting time longer than 6 months negatively influenced post-operative satisfaction and patient-related outcome because of a lower level of functional reserves and mental health [32]. In such cases, we believe that LSR could also reflect the negative influence brought by the long waiting period, which has been reported to cause a significant deterioration in quality of life and high psychological distress [33]. Once the scores of LSR are low, particularly less than or equal to 10, preoperative mental intervention is necessary to reduce the dissatisfaction resulting mainly from psychological issues. This tool may enable surgeons to evaluate the risks and benefits of surgery in each case and be useful on patient selection.

Some of the limitations of this study need to be acknowledged. Firstly, mental factors of patients are much more complex, which makes it difficult to assess them thoroughly via questionnaires, and LSR does not cover all mental aspects. Future studies should focus on overcoming these problems. Secondly, the sample size in this study was limited, and the length of 1-year follow-up period is relatively short. Thirdly, there is no classification for the preoperative radiological findings of osteoarthritis, and some studies have shown poor results in patients undergoing TKA with minimal osteoarthritis. And lastly, in this study, mental and physical changes were compared by WOMAC and SF-36, which may not be the best tools to evaluate individual outcomes.

## Conclusion

In conclusion, our study revealed that preoperative assessment of LSR can help clinicians evaluate whether a patient’s current mental status is favorable enough for a patient to accept a TKA surgery. A psychological intervention should be recommended before a TKA procedure performing if the patients were evaluated with extreme low LSR scores. This would also allow surgeons to assess the risks and benefits of surgery individually and facilitate patient selection.

## Data Availability

The dataset supporting the conclusions of this article is available on request—please contact the corresponding author Dr. Guodong Li and Dr. Pengfei Zan. Administrative permission was received from the Shanghai Tenth People’s Hospital affiliated to Tongji University to access the medical records.

## Abbreviations

TKA: Total knee arthroplasty
THA: Total hip arthroplasty
LSR: Life Satisfaction Rating
VAS: Visual analog scale
BMI: Body mass index
WOMAC: The Western Ontario and McMaster Universities Osteoarthritis Index
PCS: Physical component summary
MCS: Mental component summary
SF-36: 36-item short form surgery instrument
